# Microarray data analysis reveals gene expression changes in response to ionizing radiation in MCF7 human breast cancer cells

**DOI:** 10.1186/s41065-020-00151-z

**Published:** 2020-09-03

**Authors:** Jing Bai, Youzhen Luo, Shengchu Zhang

**Affiliations:** 1 Department of Gynaecology, The First College of Clinical Medical Sciences, China Three Gorges University, Yichang Central People’s Hospital, Yichang, 443000 Hubei China; 2 Department of Thyroid and Breast Surgery, The First College of Clinical Medical Sciences, China Three Gorges University, Yichang Central People’s Hospital, No. 183 Yiling Road, Wujia District, Yichang, 443000 Hubei China

**Keywords:** Breast cancer, Differentially expressed gene, Gene expression omnibus, Therapeutic gene targets, Transcriptional regulatory network

## Abstract

**Background:**

The aim of this study was to identify potential therapeutic target genes for breast cancer (BC) by the investigation of gene expression changes after ionizing radiation (IR) in BC cells. Gene expression profile GSE21748, including BC cell line MCF-7 samples at different time points after IR treatment, were downloaded from Gene Expression Omnibus. Differentially expressed genes (DEGs) were identified in different time points following IR compared with cell samples before IR, respectively. Gene ontology functions and The Kyoto Encyclopedia of Genes and Genomes pathways of the overlapping DEGs were enriched using DAVID. Transcription factor (TFs)-encoding genes were identified from the overlapping DEGs, followed by construction of transcriptional regulatory network and co-expression network.

**Results:**

A total of 864 overlapping DEGs were identified, which were significantly enriched in regulation of cell proliferation and apoptosis, and cell cycle process. We found that *FOXD1*, *STAT6*, *XBP1*, *STAT2*, *LMO2*, *TFAP4*, *STAT3*, *STAT1* were hub nodes in the transcriptional regulatory network of the overlapping DEGs. The co-expression network of target genes regulated by *STAT3*, *STAT1*, *STAT6* and *STAT2* included some key genes such as *BCL2L1*.

**Conclusion:**

*STAT1*, *STAT2*, *STAT3*, *STAT6*, *XBP1*, *BCL2L1*, *CYB5D2*, *ESCO2*, and *PARP2* were significantly affected by IR and they may be used as therapeutic gene targets in the treatment of BC.

## Background

Breast cancer (BC), one of the most common cancers among women, accounts for about 25% of all kinds of cancers [1, 2]. Mammary gland is not an important organ to maintain human life activities and BC in situ is not fatal. However, due to the loss of the characteristics of normal cells, the cells are loosely connected and easy to fall off. Once detached, free cancer cells can spread throughout the body by the blood or lymph, forming metastases that threaten life [3]. Despite great improvements in screening, diagnosis and treatment strategies, the prognosis and survival outcomes for breast cancer patients remain unsatisfactory [4].

In recent year, high-dosage ionizing radiation (IR) is considered to be an effective treatment for BC. Radiotherapy can significantly reduce local recurrence, BC specific mortality and total mortality [5, 6]. IR is radiation that carries enough energy to make the electrons in atoms or molecules of a material into a free state, thus ionizing those atoms or molecules [7]. In briefly, the idea behind IR therapy is that rapidly proliferating cancer cells are more sensitive to radiation than normal cells, which can repair themselves more quickly and maintain their normal function [8, 9]. So the goal of IR therapy is to inhibit the proliferative potential of cancer cells and lead to cell death, while reducing IR uptake by normal cells [10].

The response of cells to IR is a dynamic process from growth stagnation to apoptosis. And IR-induced cellular effects include sister chromatid exchange, pigment distortion, apoptosis, micronucleation, transformation, mutation and gene expression alteration [11]. In briefly, cancer cell apoptosis is achieved by DNA strand damage or indirect production of free radicals after received high-dosage IR, while the production of free radicals can indirectly damage DNA [12]. This damage is called repair-resistant or non-repairable [6]. The DNA damage response is generally activated when cells respond to these challenges. And the process of DNA damage response includes coordinating the transmission of DNA damage signals, triggering DNA repair and cell survival or apoptosis [13]. The cell response to IR is mediated by genes that control and regulate complex regulatory pathways [14]. Detection of changes in gene expression is an effective method to understand the mechanism of the above reaction. The research on transcriptional gene regulation of IR is mainly to understand how the human body reacts to IR and how radiation hazards develop.

In the present study, microarray data of gene expression in BC cells at different time points after IR were downloaded from Gene Expression Omnibus (GEO) database, and further differentially expressed genes (DEGs) were screened. Gene Ontology (GO) and The Kyoto Encyclopedia of Genes and Genomes (KEGG) pathways of the overlapping DEGs were analyzed. Then key transcription factor (TFs)-encoding genes were screened from the overlapping DEGs, followed by construction of transcriptional regulatory network and co-expression network, with the aim to identify potential therapeutic target genes for BC.

## Results

### Overlapping DEGs analysis

**Fig. 1:**
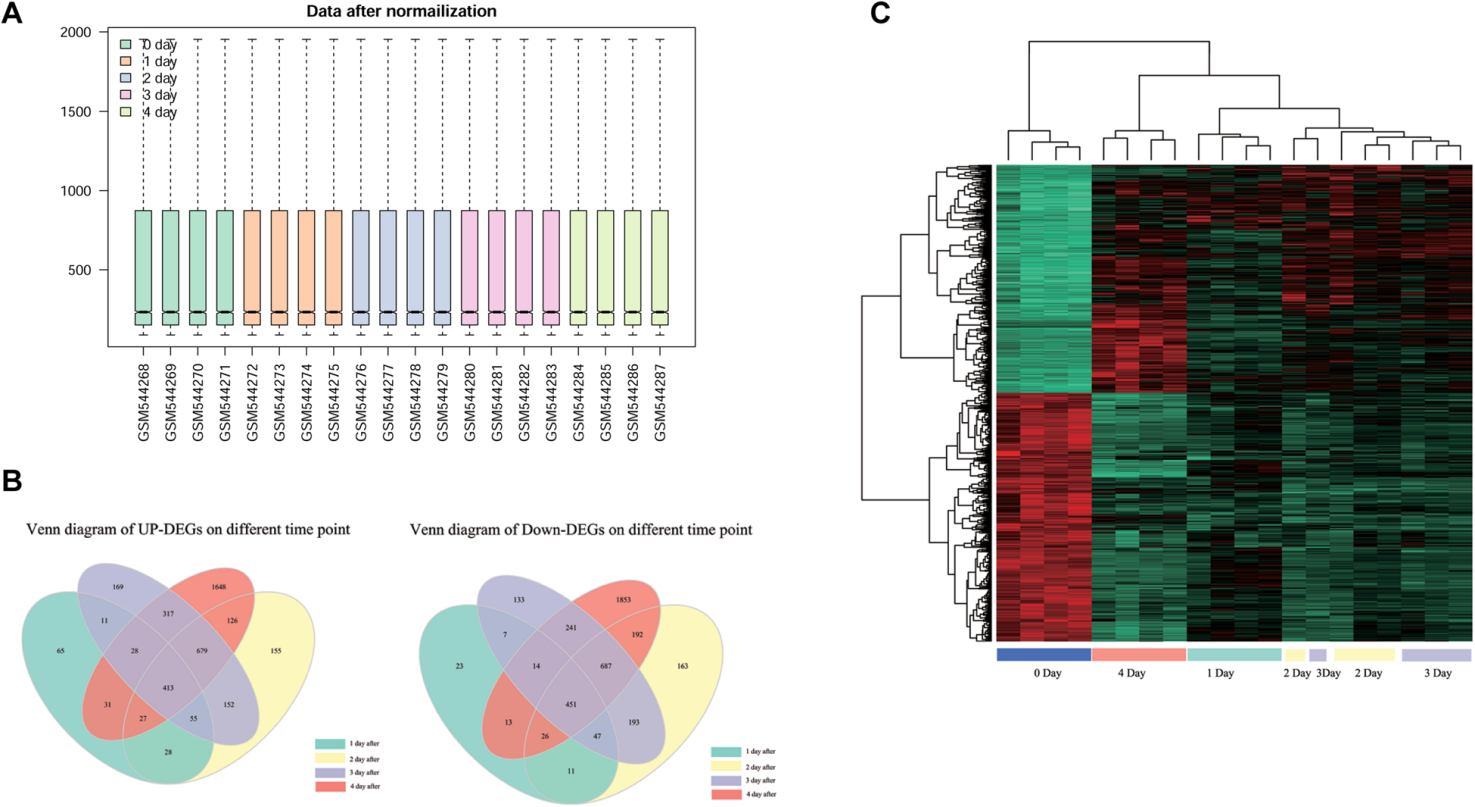
Identification of differentially expressed genes (DEGs). **a**, box plot of data after normalization. The horizontal axis refers to the sample name and longitudinal axis refers to expression values. The black line in box was the median of each set of data, through which we could determine the extend of standardization of data. The black line in box was almost in the same line which indicates good normalization. **b**, venn diagram of DEGs at different time points. **c**, heat map of overlapping DEGs. Red indicates up-regulation and green indicates down-regulation

To determine the DEGs after IR treatment in breast cancer, a microarray dataset GSE2178, obtained from MCF-7 human breast cancer cells, was downloaded from GEO. We obtained expression information of 18,179 genes from 20 samples. Box plots showed the median of expression value approximated a straight line, indicating good normalization (Fig. 1a). Totally, 1250 DEGs (592 down-regulated genes and 658 up-regulated genes) were identified after IR at day 1, 3405 DEGs at day 2 (1770 down-regulated genes and 1635 up-regulated genes), 3597 DEGs at day 3 (1773 down-regulated genes and 1824 up-regulated genes), and 6746 DEGs at day 4 (3477 down-regulated genes and 3269 up-regulated genes) compared with the control group, respectively (Table 1). Venn diagram of Up-DEGs indicated that 413 overlapping DEGs were up-regulated at four different time points (Fig. 1b). Venn diagram of Down-DEGs indicated that 451 overlapping DEGs were down-regulated at four different time points (Fig. 1b).

**Table 1:** Differentially expressed genes at different time points

Time (Day)	Number of significantly regulated genes		
	Down-regulated genes	Up-regulated genes	Total
1 Day	592	658	1250
2 Day	1770	1635	3405
3 Day	1773	1824	3597
4 Day	3477	3269	6746

From the heat map of the overlapping DEGs (Fig. 1c), we found that there was difference in DEGs expression patterns between the IR-group and control-group. The four samples on the first day post-IR or on the fourth day after radiotherapy were clustered into one category, respectively. The differences between the eight samples on days 2 and 3 were not significant. It was obviously observed that the expression of genes originally up-regulated in breast cancer began to be down-regulated after radiotherapy. It can be clearly seen from the depth of green in the Fig. 1c that the degree of down-regulation gradually deepened from the first day of the fourth day after radiotherapy. The expression level of genes originally down-regulated in breast cancer was up-regulated after radiotherapy, and the up-regulated level was significantly increased over time.

### GO and pathway enrichment analysis

In order to analyze the function of DEG and the involved pathways, DAVID was used to observe significant enrichment of these genes in multiple KEGG and GO terms. Results showed that the overlapping up-regulated DEGs were enriched in biological processes such as cell proliferation, energy metabolism and apoptosis process (Table 2); overlapping down-regulated DEGs were enriched in biological processes as cell mitosis and DNA damage repair (Table 3). Moreover, the overlapping up-regulated genes were associated with the pathways such as p53 signaling pathway, lysosome, and glutathione metabolism; the overlapping down-regulated genes were associated with the pathways such as DNA replication, cell cycle, and pyrimidine metabolism.

**Table 2:** GO and KEGG enrichment results of the up-regulated genes

Category	Term	Count	***P*** Value
GOTERM_BP_FAT	GO:0042127 ~ regulation of cell proliferation	30	0.001054887
GOTERM_BP_FAT	GO:0042981 ~ regulation of apoptosis	29	0.002919188
GOTERM_BP_FAT	GO:0043067 ~ regulation of programmed cell death	29	0.003382978
GOTERM_BP_FAT	GO:0010941 ~ regulation of cell death	29	0.003532924
GOTERM_BP_FAT	GO:0008104 ~ protein localization	26	0.048175685
GOTERM_CC_FAT	GO:0005739 ~ mitochondrion	37	0.001607912
GOTERM_CC_FAT	GO:0005829 ~ cytosol	37	0.033925941
GOTERM_CC_FAT	GO:0031090 ~ organelle membrane	35	0.006084384
GOTERM_CC_FAT	GO:0005794 ~ Golgi apparatus	30	0.004372382
GOTERM_CC_FAT	GO:0005626 ~ insoluble fraction	26	0.027666166
GOTERM_MF_FAT	GO:0046983 ~ protein dimerization activity	21	0.003932064
GOTERM_MF_FAT	GO:0042802 ~ identical protein binding	23	0.005855689
GOTERM_MF_FAT	GO:0043028 ~ caspase regulator activity	4	0.017632928
GOTERM_MF_FAT	GO:0048037 ~ cofactor binding	11	0.021981803
GOTERM_MF_FAT	GO:0042803 ~ protein homo dimerization activity	13	0.027801507
KEGG_PATHWAY	hsa04115:p53 signaling pathway	8	0.001608728
KEGG_PATHWAY	hsa04142:Lysosome	10	0.002837651
KEGG_PATHWAY	hsa05416:Viral myocarditis	7	0.009152891
KEGG_PATHWAY	hsa00480:Glutathione metabolism	5	0.037989004

**Table 3:** GO and KEGG enrichment results of the down-regulated genes

Category	Term	Count	***P*** Value
GOTERM_BP_FAT	GO:0007049 ~ cell cycle	99	3.71E-44
GOTERM_BP_FAT	GO:0006259 ~ DNA metabolic process	80	6.51E-42
GOTERM_BP_FAT	GO:0022402 ~ cell cycle process	76	9.75E-35
GOTERM_BP_FAT	GO:0022403 ~ cell cycle phase	69	2.49E-37
GOTERM_BP_FAT	GO:0000279 ~ M phase	63	1.32E-37
GOTERM_CC_FAT	GO:0043232 ~ intracellular non-membrane-bounded organelle	125	2.16E-21
GOTERM_CC_FAT	GO:0043228 ~ non-membrane-bounded organelle	125	2.16E-21
GOTERM_CC_FAT	GO:0070013 ~ intracellular organelle lumen	106	1.53E-24
GOTERM_CC_FAT	GO:0043233 ~ organelle lumen	106	9.59E-24
GOTERM_CC_FAT	GO:0031974 ~ membrane-enclosed lumen	106	4.56E-23
GOTERM_MF_FAT	GO:0003677 ~ DNA binding	92	1.81E-08
GOTERM_MF_FAT	GO:0000166 ~ nucleotide binding	91	6.03E-09
GOTERM_MF_FAT	GO:0017076 ~ purine nucleotide binding	83	1.95E-09
GOTERM_MF_FAT	GO:0032555 ~ purine ribonucleotide binding	82	5.64E-10
GOTERM_MF_FAT	GO:0032553 ~ ribonucleotide binding	82	5.64E-10
KEGG_PATHWAY	hsa03030:DNA replication	22	1.63E-25
KEGG_PATHWAY	hsa04110:Cell cycle	26	1.22E-16
KEGG_PATHWAY	hsa00240:Pyrimidine metabolism	13	3.29E-06
KEGG_PATHWAY	hsa03430:Mismatch repair	12	1.60E-12

### Construction of transcriptional regulatory network

**Fig. 2:**
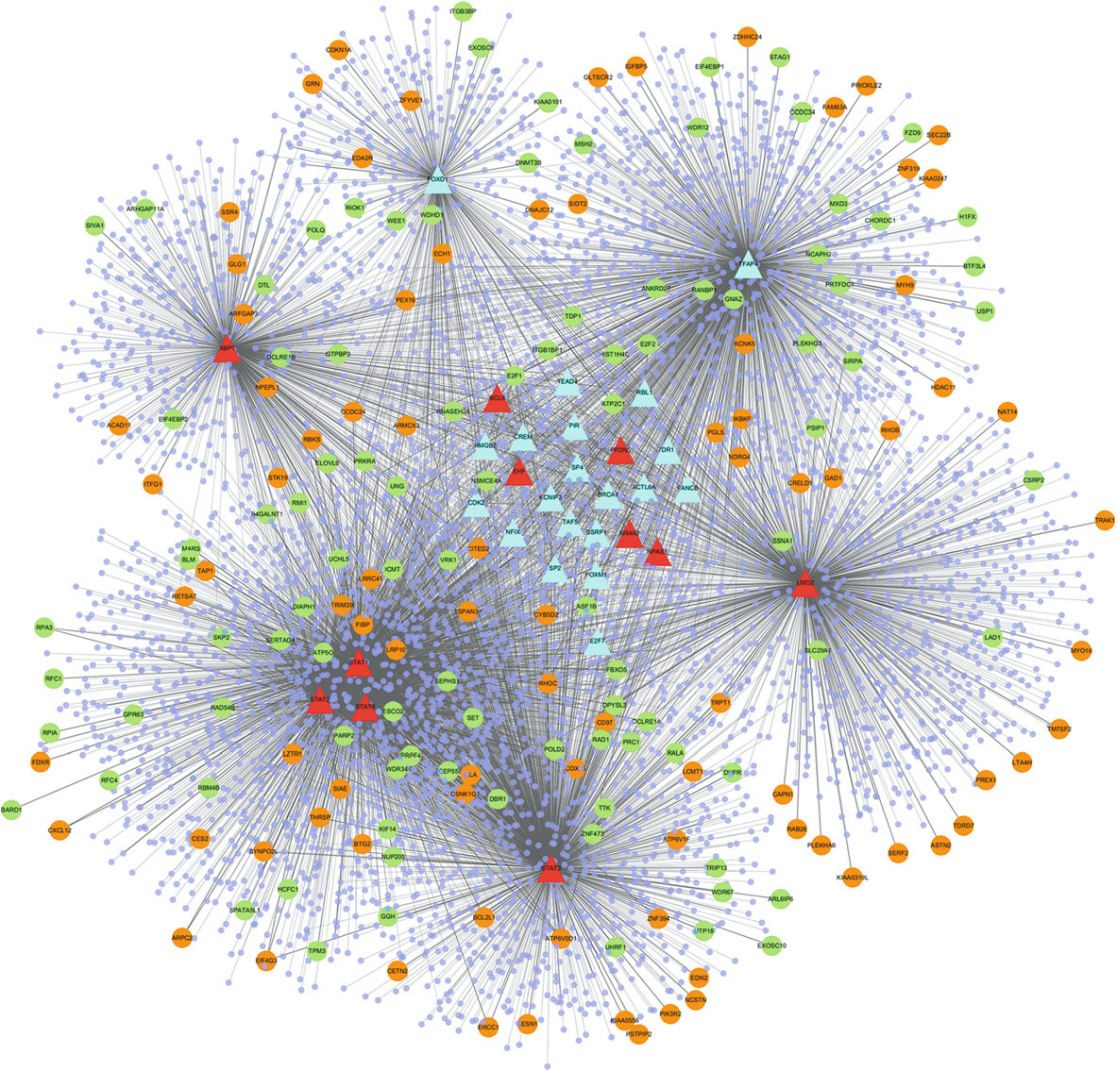
Transcriptional regulatory network of transcription factors (TFs) and target genes. Triangle nodes refer to corresponding genes of TFs, dots to the target genes regulated by TFs, purple dots that do not give name of genes were differentially expressed genes which were not focused on in this study, the green dots to down-regulated genes, orange dots to up-regulated genes, the red triangles to up-regulated TFs coding genes, blue triangles to down-regulated TFs coding genes

**Fig. 3:**
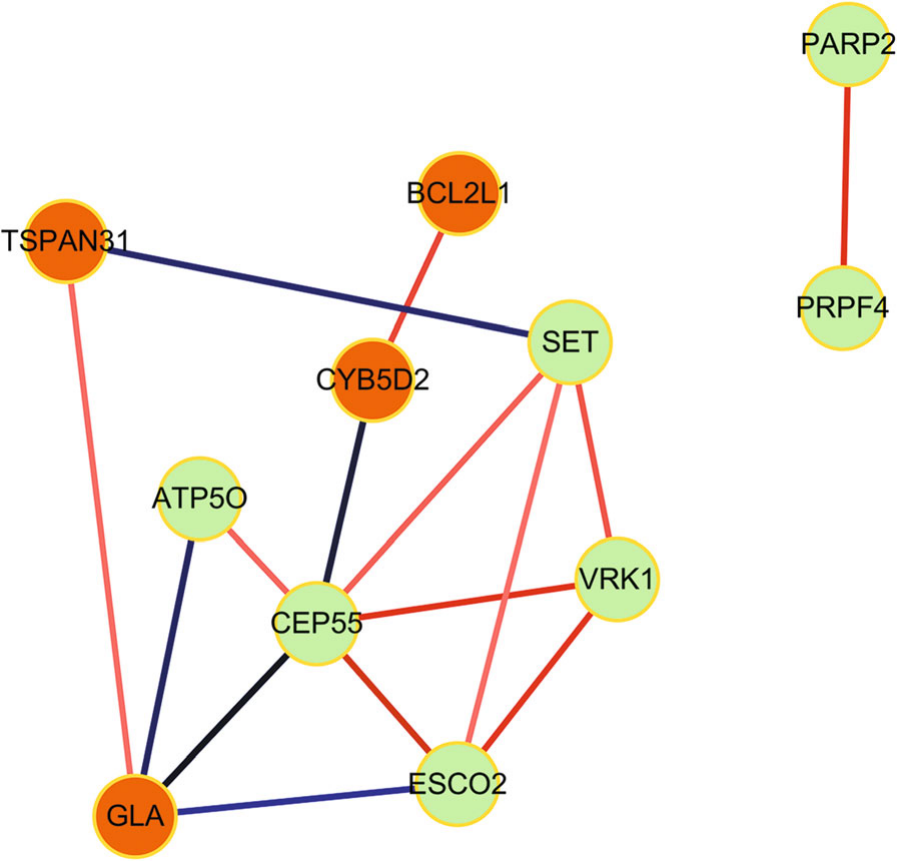
Co-expression network of DEGs regulated by STAT1, STAT2, STAT3 and STAT6. Edge refers to absolute value of similarity coefficient of the expression of two genes in different samples was greater than 0.85 and *p* value < 0.05, the red edge represented the positive correlation, green edge represented negative correlation, orange node represented up-regulated genes, green nodes for down-regulated genes

A total of 12 genes that encoded TFs were found in the overlapping up-regulated DEGs, and 24 genes that encoded TFs were identified in the overlapping down-regulated DEGs. According to the transcriptional regulatory network which was arranged in a radial pattern, we found that 8 of the 36 TF encoding genes regulated more target genes, including Forkhead Box D1 (FOXD1, down-regulated, degree of connectivity = 444), Signal Transducer And Activator Of Transcription 6 (STAT6, down-regulated, degree of connectivity = 587), X-Box Binding Protein 1 (XBP1, down-regulated, degree of connectivity = 806), STAT2 (down-regulated, degree of connectivity = 948), LIM Domain Only 2 (LMO2, down-regulated, degree of connectivity =1130), Transcription Factor AP-4 (TFAP4, down-regulated, degree of connectivity = 1584), STAT3 (down-regulated, degree of connectivity = 1635), and STAT1 (Signal Transducer And Activator Of Transcription 1, down-regulated, degree of connectivity =1968) (Fig. 2). Then we screened the target genes in the overlapping DEGs regulated by STAT1, STAT3, STAT2 and STAT6, and 14 genes were obtained, which were then subjected to the co-expression network construction (Fig. 3). Genes in the co-expression network included BCL2 Like 1 (*BCL2L1*), Cytochrome B5 Domain Containing 2 (*CYB5D2*), Tetraspanin 31 (*TSPAN31*), Galactosidase Alpha (*GLA*), ATP Synthase Peripheral Stalk Subunit OSCP (*ATP5O*), SET Nuclear Proto-Oncogene (*SET*), Centrosomal Protein 55 (*CEP55*), Vaccinia Related Kinase 1 (*VRK1*), Establishment Of Sister Chromatid Cohesion N-Acetyltransferase 2 (*ESCO2*), Poly (ADP-Ribose) Polymerase 2 (*PARP2*) and Pre-MRNA Processing Factor 4 (*PRPF4*).

## Discussion

In this study, bioinformatics approach was used to predict potential therapeutic targets for BC. We have identified 864 overlapping DEGs between IR groups and control group, among which 413 genes were down-regulated and 451 ones were up-regulated. By constructing transcriptional regulatory network, we found several key hub nodes including STAT3, STAT6, XBP1, STAT2 and STAT1. Moreover, co-expression network on the genes regulated by STAT3, STAT6, STAT2 and STAT1 was constructed.

Studies showed that target genes regulated by STAT proteins were important in cancers [15–17]. STAT family proteins regulate the expression of a variety of genes involved in cell growth, survival, differentiation and apoptosis [18, 19]. A total of seven STAT proteins have been identified, including STAT1 2, 3, 4, 5a, 5b, and 6 [18, 19]. Studies have shown that some members of the STAT family are tumor suppressor genes of BC, while others are oncogenes [20, 21]. Kolla et al. found that STAT1 was tumor suppressors and lack of their expression may be involved in tumorigenesis [22]. Jung et al. showed that STAT1 are downstream targets in MCF-7 human BC cell and have tumor suppressor function in BC [23]. Gooch et al. found that STAT6 mediates the inhibition of interleukin-4 growth in human BC cells [24]. Studies found that STAT3 was an oncogene and over expressed in BC [25]. Behera et al found that osteopontin can promote tumor growth of human BC cells by activating the JAK2/STAT3 signaling pathway [26]. Bharadwaj et al. found that many biological processes were regulated by STAT3, including cancer cell growth, apoptosis resistance, and DNA damage response. Moreover, STAT3 has also been proved to be the target of tumor therapy [27]. Wei et al. showed that STAT6 was required for the inhibition of BC cell growth [28]. In our study, according to the result of GO and pathway analysis, we found that STAT1 STAT2, STAT3, and STAT6 may be involved in the biological processes like cell proliferation, apoptosis and programmed cell death. Therefore, we speculated that STAT1 STAT2, STAT3 and STAT6 could be therapeutic targets for BC.

Among the key hub nodes there was also XBP-1, one kind of basic region leucine zipper protein, which has reported having a high expression level Estrogen receptor alpha (ERα)-positive breast tumors [29–32]. ERα has been a primary target of treatment as well as a prognostic indicator for BC [33]. Studies have found that the transcription level of XBP-1 is related to ERα and can increase the transcriptional activity of ERα. This process is achieved by regulating the chromatin unfolding in BC [33, 34]. Furthermore, some studies showed that activation of ERα is responsible for many biological processes, including cell growth and differentiation and programmed cell death [32, 35]. Overexpression of XBP-1 has been identified in primary BC [36] while XBP-1 was down-regulated after radiotherapy treatment. Thus, we suggested that XBP-1 may be a key regulator underlying the development of BC.

Among the genes significantly affected by ionizing radiation, we discovered BCL2L1, a gene involved in the regulation of apoptosis [37], and CYB5D2 that inhibits cell proliferation and has a putative tumor suppressor activity [38]. Anthony et al. suggested that CYB5D2 was an inhibitor of cell proliferation and had putative tumor suppressor activity [39]. BCL2L1 and CYB5D2 were all in the CpG island, indicating lower expression of them may be caused by the methylation that leading to gene silencing. DNA methylation, which primarily occurs at CpG sites plays an important role in transcriptional regulation and tumor initiation [40, 41]. Overall, *BCL2L1* and *CYB5D2* could be attractive targets for future BC therapies.

VRK1, ESCO2, and PARP2 were included in the co-expression network (Fig. 3). Thereinto, VRK1 as proliferation-promoting nuclear kinase has been reported to play a role in cell migration and invasion. Overexpression of *VRK1* can promote a mesenchymal to epithelial transition (MET) in cell culture, while VRK1-mediated MET might facilitate the colonization of distal sites by metastatic BC cells [42]. Additionally, the effect of *VRK1* to protect against DNA damage was determined by studying the effect of its knockdown on the formation of DNA repair in response to treatment with IR in BC cell line [43]**.** ESCO2 has been described as a regulator of mitosis and required for DNA damage repair [44, 45]. As a mitosis regulator, ESCO2 could uniquely promote cohesion between sister chromatids [46]. GO analysis showed that *PARP2* and *ESCO2* mainly involved in the metabolic process of DNA and could respond to DNA damage repair. All the results suggested that *VRK1*, *ESCO2* and *PARP2* might participate in the process of resistant BC and could be used as new drug targets since these genes may contribute to cell protection against DNA damage.

## Conclusions

In conclusion, the *STAT1*, *STAT2*, *STAT3*, *STAT6*, *XBP1*, *BCL2L1*, *CYB5D2*, *ESCO2*, and *PARP2* may be an important component in the progression and development of BC. As potential specific targets for the treatment of BC, they may play an important role. Meanwhile, necessary clinical validation trials are also a key part of verifying the accuracy of these potential therapeutic targets.

## Methods

### Affymetrix microarray data

Based on the platform of GPL6104 (Illumina humanRef-8 v2.0 expression beadchip) which was deposited in GEO (http://www.ncbi.nlm.nih.gov/geo/), the expression profile GSE21748 was obtained [47]. MCF-7 samples in this study were exposed to γ-ray with a ^137^Cs γ-ray source at a dose rate of 3.0 Gy/min. Samples which were treated with IR after 1 day, 2 day, 3 day, 4 day and no treatment (0 day) were collected. Each time point included 4 repeat samples. We also downloaded the raw data and annotation files from GEO database.

Probes with missing expression values were removed, the probe name was converted to gene name using the platform annotation information, and the average expression values of different probes that corresponded to the same gene were considered as the expression value of this gene. In addition, the data were normalized using the median method and the data distribution was displayed using box plots. Then, the DEGs between IR-group and control-group (day0) were analyzed by limma package in R [48]. Using Beniamini-Hochberg false discovery rate (FDR), the multiple testing correction was performed [49]. The DEGs with adjusted *p*-value < 0.05 and |log fold change (FC)| > 2 were considered to be significant.

### Overlapping DEGs analysis

Venn diagrams of up-DEGs and down-DEGs at different time points were performed, respectively. The common DEGs were selected. Based on the probe information in the download file, the expression value of each group’s overlapping data was selected. Based on the Euclidean distance, the R language pheatmap package (http://cran.r-project.org/web/packages/pheatmap/index.html) was used for Hierarchical clustering [50] of the overlapping DEGs [51]. The heat map was used as the result display. Using Duplexing cluster, genes having similar expression levels were collected together for a further research.

### Gene ontology (GO) and pathway enrichment analysis

In this study, GO function and Kyoto Encyclopedia of Genes and Genomes (KEGG) pathway of the overlapping DEGs were analyzed using The Database for Annotation, Visualization and Integrated Discovery (DAVID, version 6.8, https://david.ncifcrf.gov/) with the threshold of *p*-value < 0.05.

### Transcriptional regulatory network analysis

The genes encoding TFs were screened from the collection of the overlapping DEGs based on the information provided by Transcription Factor Database (TRANSFAC) [52]. The interactions between target genes and transcription factors (TFs) were predicted using TF binding sites information from the UCSC database [53]. Using Cytoscape, the transcriptional regulatory network was constructed [54]_._ The calculation of the connectivity of TF nodes, the selection of key TF and key target genes was carried out by using Igraph package. In addition, functional analysis of key genes was performed in combination with expression trend information. Then, we selected target genes regulated by significant TFs from the overlapping DEGs, and calculated the correlation coefficient between them. The co-expression gene pairs with Pearson similarity coefficient > 0.85 and *p*-value < 0.05 were selected and used to build the co-expression network.

## Data Availability

The datasets used and/or analyzed during the current study are available from the corresponding author on reasonable request.
